# Rice Cytochrome P450 Protein CYP71P1 Is Required for Heat Stress Tolerance by Regulating Serotonin Biosynthesis and ROS Homeostasis

**DOI:** 10.3390/plants14071072

**Published:** 2025-04-01

**Authors:** Xuantong Lv, Xunan Zhao, Fang Wang, Haili Wang, Yanli Zhang, Banpu Ruan, Guojun Dong, Yanchun Yu, Limin Wu, Fei Chen

**Affiliations:** 1 College of Life and Environmental Sciences, Hangzhou Normal University, Hangzhou 311121, China; 2022111010019@stu.hznu.edu.cn (X.L.); zhaoxunanl@163.com (X.Z.); hail_117@163.com (H.W.); zhangyanli1121@sina.com (Y.Z.); ruanbp123@163.com (B.R.); ycyu@hznu.edu.cn (Y.Y.); 2 Institute of Insect Sciences, Zhejiang University, Hangzhou 310058, China; wangf121@zju.edu.cn; 3 State Key Laboratory for Rice Biology, China National Rice Research Institute, Hangzhou 310006, China; guojd@hotmail.com

**Keywords:** *HTS2*, Heat tolerance, rice (*Oryza sativa*), ROS homeostasis, serotonin

## Abstract

Heat stress is one of the major factors affecting crop growth and yield. However, the molecular mechanisms underlying rice heat stress tolerance remain largely unclear. In this study, we identified and characterized the rice *high temperature sensitive 2* (*hts2*) mutant, which is highly susceptible to heat stress. Map-based cloning revealed that the *HTS2* encodes a cytochrome P450 protein (CYP71P1) involved in serotonin biosynthesis. *HTS2* is ubiquitously expressed across plant tissues and shows strong upregulation in response to heat stress. The *HTS2* mutation significantly impairs basal serotonin synthesis in rice, and the heat-sensitive phenotype of the *hts2* mutant is completely rescued by exogenous serotonin supplementation. Compared to the wild type, the *hts2* mutant exhibits reduced antioxidant capacity, leading to excessive reactive oxygen species (ROS) accumulation and severe oxidative damage, ultimately reducing heat stress tolerance. Furthermore, disruption of *HTS2* significantly affects the rice heat shock response, with the heat-induced expression of *HsfA2s* and their downstream target genes, such as *HSP18.0* (*heat shock protein 18.0*) and *OsAPX2* (*ascorbate peroxidase 2*), markedly depressed in *hts2* mutant. Our results suggest a pivotal role of *HTS2* in modulating serotonin metabolism and maintaining ROS homeostasis during heat stress, offering new perspectives on the mechanisms underlying heat tolerance and potential strategies to enhance rice resilience to heat stress.

## 1. Introduction

Frequent heat waves associated with global warming have emerged as a significant abiotic stressor for crop growth and development, leading to declining yields and posing a considerable threat to global food security [1]. Rice, a staple cereal crop for nearly half the global population, is particularly susceptible to heat stress. Developing stress-resilient rice varieties is imperative to combat the growing challenge of food insecurity, which relies on a deeper understanding of the molecular mechanisms underlying plant heat tolerance [2]. Research in *Arabidopsis* has revealed that plant heat stress responses involve complex gene regulatory networks, including heat sensing, signal transduction, transcriptional, and translational regulation [3,4,5]. Among these, heat shock factors (HSFs) and heat shock proteins (HSPs) are central to the acquisition of heat stress tolerance. Under heat stress, HSFs are rapidly upregulated and bind to heat shock elements (HSEs) in the promoters of downstream target genes, thereby modulating their transcriptional activity [6,7]. In *Arabidopsis*, HsfA1 serves as a master regulator of heat shock response (HSR) [8]. Heat stress activates HsfA1 by relieving the inhibitory effects of Hsp70 and Hsp90, leading to the induction of downstream transcription factors such as HsfA2, Dehydration-Responsive Element Binding Protein 2A (DREB2A), and Multiprotein Bridging Factor 1C (MBF1C). These transcription factors, in turn, orchestrate the expression of heat stress-inducible genes, thereby enhancing heat stress tolerance [9,10]. Additionally, the heat-shock transcriptional response depends on various signal transduction pathways, including calcium ions (Ca^2+^), reactive oxygen species (ROS), nitric oxide (NO), phospholipid signaling, and phytohormones [11,12,13]. While the molecular mechanisms governing heat stress response in *Arabidopsis* are well-characterized, the regulatory pathways underlying heat stress tolerance in rice are still poorly understood.

Heat stress can induce a series of physiological and biochemical changes in rice cells. One direct consequence of these cellular alterations is the accumulation of toxic compounds that include ROS, such as hydrogen peroxide (H_2_O_2_), superoxide (O_2_^−^), and hydroxyl radical (HO·) [14]. Heat-induced ROS burst acts as early signals enabling cells to rapidly respond to heat stress [12]. However, excessive ROS accumulation induced by continuous heat stress can result in irreversible cellular oxidative damage, including membrane lipid peroxidation, DNA damage, protein denaturation, chloroplast degradation, and ultimately, cell death [15,16,17]. Therefore, maintaining low cellular ROS accumulation is critical for the induction of heat stress acclimation. In plants, cellular ROS are tightly regulated by a robust antioxidant defense system comprising various enzymatic and non-enzymatic antioxidants [18]. Research has demonstrated that maintaining strong ROS scavenging ability in cells significantly improves heat stress tolerance in rice. For instance, Semi-Rolled Leaf 10 (SRL10) and Enhanced Disease Susceptibility 1 (EDS1) regulate rice heat stress tolerance by interacting directly with and enhancing catalase (CAT)-mediated H_2_O_2_ scavenging activities [19,20]. OsSNAC3, a stress-responsive NAC transcription factor, regulates ROS balance by controlling the expression of antioxidant enzyme genes, thereby enhancing heat tolerance in rice [21]. Moreover, the Ca^2^⁺ signal cascade also contributes to the regulation of heat-induced cellular ROS homeostasis. Heat stress-induced H₂O₂ production upregulates the expression of *OsANN1*, a calcium-binding annexin in rice, which boosts the activities of superoxide dismutase (SOD) and CAT, thereby maintaining ROS homeostasis and ultimately enhancing heat stress tolerance [22]. Conversely, loss-of-function mutations in OsCNGC14 and OsCNGC16, two cyclic nucleotide-gated ion channel (CNGC) proteins, lead to heat sensitive phenotypes due to aberrant heat-induced Ca^2+^ influx and ROS accumulation [23]. In addition, the knockout of *OsRbohB*, an NADPH oxidase gene involved in ROS production, markedly reduces heat-induced ROS accumulation and enhances heat stress tolerance in rice [24]. These studies highlight the critical importance of finely tuned ROS balance for plants to effectively respond and adapt to heat stress conditions.

Serotonin (5-hydroxytryptamine), a pineal hormone first identified in mammals, has been demonstrated to function as a pivotal regulator in plant growth and stress adaptation [25]. In plants, the biosynthesis of serotonin is closely mediated by 2-tryptophan decarboxylase (TDC) and tryptamine 5-hydroxylase (T5H), which sequentially convert tryptophan to serotonin [26]. Serotonin can subsequently be transformed into melatonin through a series of enzymatic reactions [27]. Previous studies have shown that both serotonin and melatonin play significant roles in enhancing plant stress tolerance [25]. Abiotic stressors such as salt and cadmium can markedly elevate the serotonin and melatonin biosynthesis in different plant species [28,29,30]. Meanwhile, exogenous application of serotonin and melatonin has been reported to effectively alleviate stress-induced cellular damage. For example, improved tolerance to salt and drought stresses due to serotonin and melatonin supplement has been demonstrated in different plant species [29,31]. Further investigations reveal that these metabolites can alleviate stress damage by promoting plant growth and enhancing antioxidant systems [32]. On the other hand, a reduction in endogenous serotonin content significantly impairs the protective effects induced by cold acclimation under cold stress in *Kandelia obovata* [33]. These findings provide further support for the involvement of serotonin in mediating plant stress responses [34,35]. However, the mechanism of serotonin action in plant heat stress acclimation processes remain unclear. In this study, we report the identification and characterization of *High Temperature Sensitive 2* (*HTS2*), which encodes a previously reported cytochrome P450 superfamily protein (CYP71P1) involved in serotonin biosynthesis. The mutation of *HTS2* significantly impacts the accumulation of endogenous serotonin, leading to excessive ROS accumulation and ultimately plant death under heat stress. Our results suggest that *HTS2*-dependent serotonin biosynthesis is critical for heat stress tolerance through maintaining oxidative balance and mediating heat shock response in rice. Investigating *HTS2* will enhance our understanding of serotonin’s role and its underlying mechanisms in regulating plant stress adaptation.

## 2. Results

### 2.1. hts2 Mutant Is Hypersensitive to Heat Stress

The *hts2* mutant was obtained from an EMS-induced mutagenized population of the *Indica* rice cultivar, Shuhui 527. Under field growth conditions, *hts2* mutant plants exhibited leaf lesion mimic and early senescence phenotypes (Figures S1 and S2). The spontaneous lesions began to appear on the blade edge of the *hts2* mutant at the late seedling stage, which continued to spread and became more obvious as the plant grew (Figure S1A–C). Histochemical analysis revealed more H_2_O_2_ and O_2_^−^ accumulation in the lesion regions (Figure S1D). By the maturing stage, the *hts2* mutant displayed a typically early senescence phenotype, associated with rapidly elevated transcript levels of several senescence-related genes (e.g., *PS1*, *NYC3*, *SGR*, *Osh36,* and *OsI58*; Ref. [36]) compared to the WT (Figure S2A,B). Consistent with this, *HTS2* deficiency could significantly accelerate dark-induced leaf senescence (Figure S2C,D). In addition, we also found some mild morphological defects, such as slightly decreased plant height, shorter panicles, fewer primary panicles, and smaller grain size. However, no significant difference in 1000-grain weight was observed between the *hts2* mutant and wild-type plants (Figure S1E–G).

**Figure 1:**
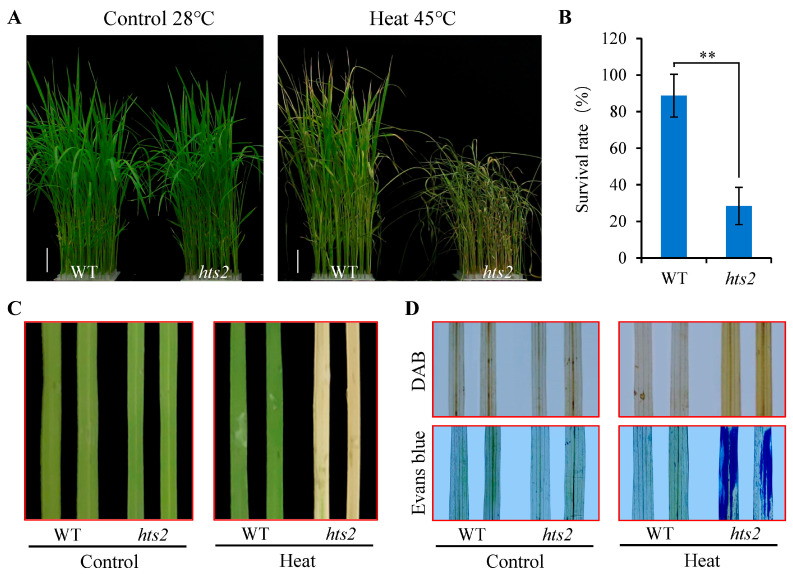
*hts2* mutants exhibit heat stress-sensitive phenotypes. (**A**) Phenotypes of the wild type (WT) and *hts2* mutant seedlings before and after heat treatments. Two-week-old rice seedlings grown at normal temperature (28 °C) were transferred at 45 °C for 3 days and then recovered at 28 °C for 2 days. Scale bar, 2 cm. (**B**) Survival rates of WT and *hts2* mutant seedlings after heat treatment and recovery. Data are means ± SD (n = 3 replicates, 30–40 individual seedlings per replicate). Asterisks indicate significant difference between the WT and mutant by Student’s *t*-tests (** *p* < 0.01). (**C**) Heat-sensitive phenotype of *hts2* mutant was examined with detached leaf. (**D**) Visualization of H_2_O_2_ accumulation (top panel) and cell death (lower panel) using DAB and Evans blue staining of the heat-challenged detached leaves as described in (**C**).

Moreover, the *hts2* mutant displayed reduced heat tolerance at the seedling stage. Under normal growth conditions (28 °C), no notable phenotypic differences were observed between the wild type (WT) and *hts2* mutant seedlings. However, the *hts2* mutant showed a more sensitive heat shock phenotype compared to the WT, as indicated by earlier leaf curling and wilting and dramatically reduced survival rates (*hts2* vs. WT; 28.5% vs. 88.7%) when subjected to 45 °C heat treatment (Figure 1A,B). The heat-sensitive phenotype of the *hts2* mutant was further confirmed by detached leaf assay. After 5 h heat treatment and 3 d recovery under light, the color of detached leaves from the *hts2* mutant almost turned white, whereas the majority of WT leaves remained green (Figure 1C). Moreover, compared with the WT, *hts2* detached leaves had considerably more ROS and dead cell accumulation (Figure 1D).

### 2.2. Heat Treatment Accelerated Lesion-Mimic Symptoms Formation in hts2 Mutant

**Figure 2:**
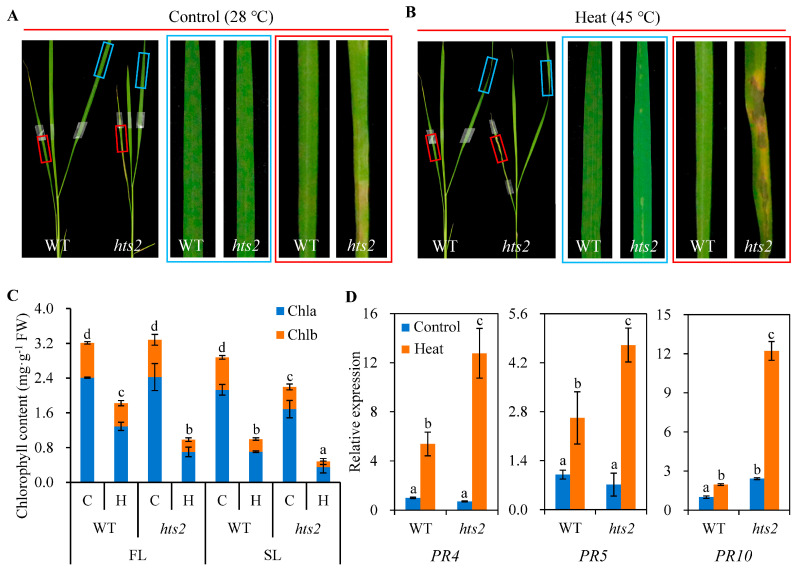
*HTS2* deficiency accelerates the development of lesion-mimic symptoms under heat stress conditions. (**A**,**B**) Leaf phenotypes of the wild type (WT) and *hts2* mutant plants treated at different temperature conditions. Three-week-old seedlings grown at 28 °C were used for treatments. Enlarged views of the red and blue boxes areas of the representative leaves of WT and *hts2* are shown right. Blue and red boxes indicate the first (FL) and second (SL) fully expanded leaves from the top of the plant, respectively. (**C**) Chlorophyll contents of leaves from WT and *hts2* mutant before and after heat treatment. Chla, Chlorophyll a; Chla, Chlorophyll b; C, Control; H, Heat. (**D**) Relative expression levels of several pathogenesis-related genes (*PRs*) in leaves of WT and *hts2* mutant plants exposed to 28 °C or 45 °C for 3 h at the seedling stage. Data are means ± SD (n = 3). Different lowercase letters above the error bars indicate a significant difference at *p* < 0.05 by Duncan’s multiple range test. FW, fresh weight.

Since temperature is one of the important factors to induce lesion mimic symptoms [17], we then investigate whether the lesion formation in the *hts2* mutant is affected by heat treatment. Three-week-old WT and *hts2* mutant seedlings were grown in controlled chambers under either normal (28 °C) or high temperature (45 °C) conditions. Both the *hts2* and WT seedlings growing under normal temperature condition did not display local lesion (Figure 2A). However, high temperature treatment significantly enhanced the lesion formation and development in the *hts2* mutant. After five days of heat treatment, small mosaic lesions appeared on the new leaves (the first fully expanded leaves from the top) of the *hts2* mutant, while old leaves (the second fully expanded leaves from the top) exhibited larger and more numerous lesions that frequently coalesced. In contrast, no lesions were observed on the leaves of WT plants (Figure 2B). Meanwhile, we observed that heat stress significantly accelerated leaf chlorosis in the *hts2* mutant. After five days of heat treatment, the contents of chlorophyll (Chl) a and b, as well as the Chl a/b ratios, in both new and old leaves of *hts2* seedlings were markedly reduced compared to those in the WT plants (Figure 2C). Lesion development in rice lesion-mimic mutants are often concomitant with constitutive elevated expression of defense response genes [37]. We also found that several pathogenesis-related protein genes, including *OsPR4*, *OsPR5,* and *OsPR10*, were significantly up-regulated in *hts2* mutant (Figure 2D). These results suggest that heat stress can precipitate the emergence of lesion-mimic symptoms and accelerate the progression of this symptom in the *hts2* mutant.

### 2.3. Map-Based Cloning of HTS2

**Figure 3:**
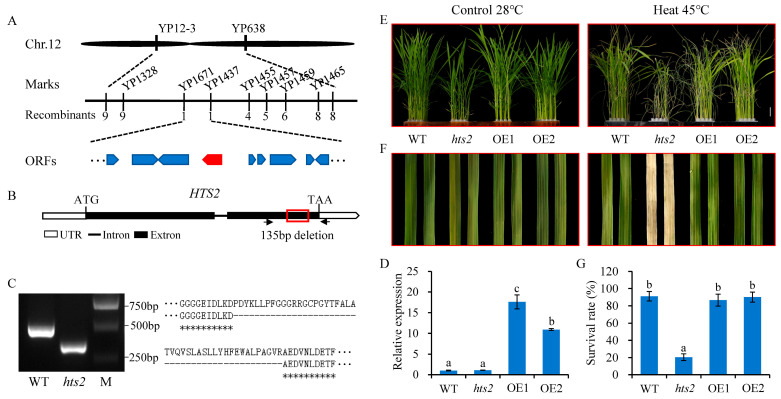
Map-based cloning and verification of *HTS2* function. (**A**) Map-based cloning of the *HTS2* gene. The *HTS2* locus was initially mapped to chromosome 12 (Chr. 12) between markers 12-3 and yp638 and further refined to a 192-kb region between yp1437 and yp1671 using a large F2 mapping population. (**B**) Structure of the *HTS2* gene. The 135 bp deletion in *hts2* is indicated (red box). (**C**) RT-PCR results showing a 135-bp deletion in the mRNA of *hts2*. A 400-bp DNA fragment spanning the mutation was amplified using specific primers (YP6087, indicated by black arrow in (**B**)). The alignment of protein sequences from WT and *hts2* mutant is shown on the right. Identical residues are marked with asterisks. (**D**) qRT-PCR analysis of *HTS2* expression in leaves of the wild type (WT), *hts2,* and *HTS2* overexpression (OE) lines. (**E**) Morphology of 2-week-old seedlings of WT, *hts2,* and OE lines grown at 28 °C, after 72 h at 45 °C, and following 2 days recovered at 28 °C. (**F**) Images of the detached leaves from four different genotypes described in (**E**) after heat stress. (**G**) Survival rates of WT, *hts2,* and OE lines after 72 h of heat treatment at 45 °C. Data are means ± SD (n = 3 replicates, 30–40 seedlings per replicate). Different lowercase letters above the error bars indicate a significant difference at *p* < 0.05 by Duncan’s multiple range test.

To identify the gene associated with the mutant phenotype, an F2 population was created by crossing *hts2* with Nipponbare. Segregation analysis indicated that the mutant phenotype is governed by a single recessive gene. By using 20 F2 recessive individuals from the mapping population and employing 180 PCR-based molecular markers evenly distributed across the 12 rice chromosomes, the *HTS2* locus was initially localized to a region between the markers YP12-3 and YP638 on chromosome 12 (Figure 3A). Subsequently, another 285 resistant F2 individuals and seven new developed STS markers were used for fine mapping, and the *HTS2* locus was narrowed to a 192-kb interval flanked by two STS markers YP1437 and YP1671 (Figure 3A). According to data from the Rice Genome Annotation Project, this region includes 18 annotated candidate open reading frames (ORFs). Sequence analysis of the *hts2* mutant revealed a 135 bp nucleotide deletion in Exon 2 of *LOC_Os12g16720*, resulting in 45 amino acids deletion (Figure 3B,C).

To conclusively verify that the *HTS2* mutation causes the observed phenotype, an expression plasmid containing the entire coding region of *LOC_Os12g16720* under the control of the Actin promoter was constructed and introduced into the *hts2* mutant via *Agrobacterium*-mediated transformation. qRT-PCR analysis confirmed a significant increase in *HTS2* transcript levels in two independently transgenic lines (Figure 3D). Phenotypic observations showed that transgenic lines displayed phenotypes identical to WT plants under field conditions (Figure S3). When subjected to high temperature conditions, the transgenic lines grew and responded to heat stress normally, comparable to the WT (Figure 3E–G). To further confirm that *LOC_Os12g16720* is the corresponding *HTS2* gene, we generated 10 transgenic *HTS2*-knockdown lines in the Nipponbare background using RNA interference (RNAi) technology. Compared with Nipponbare, the two independently RNAi lines with significant reduction in *HTS2* expression exhibited leaf lesion mimic, early senescence, and high temperature sensitivity phenotypes (Figure S4), similar to our observations in *hts2* mutant plants. These findings confirm that *LOC_Os12g16720* corresponds to the *HTS2* gene.

**Figure 4:**
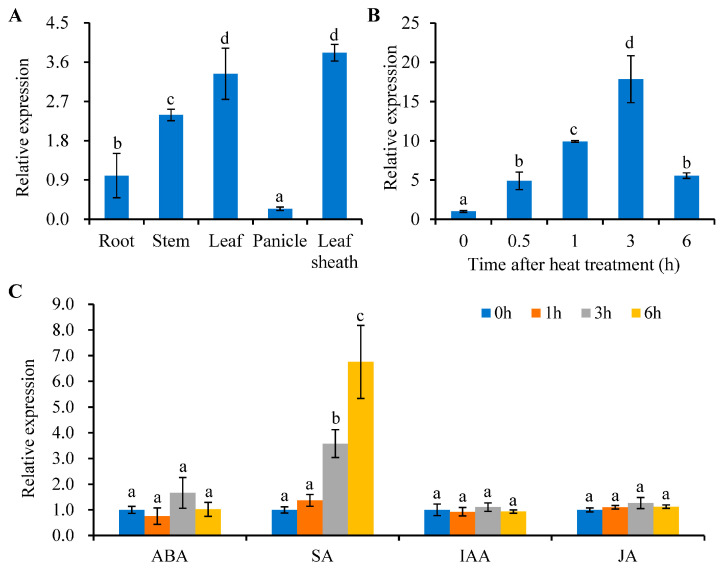
Expression pattern of *HTS2*. (**A**) qRT-PCR analysis of *HTS2* expression in various tissues, including roots, leaves, leaf sheaths, stems, and young panicles. (**B**) Transcription levels of *HTS2* under 45 °C heat treatment for the indicated time. (**C**) *HTS2* expression in response to different plant hormone treatments. Two-week-old plants were treated with ABA (10 µM), SA (500 µM), IAA (10 μM), or MeJA (50 µM) for the indicated times. Data are means ± SD (n = 3). Different lowercase letters above the error bars indicate a significant difference at *p* < 0.05 by Duncan’s multiple range test.

As evidenced by qRT-PCR analysis, *HTS2* is expressed in multiple plant tissues, including roots, leaves, leaf sheaths, stems, and panicles. The highest expression levels are observed in leaves and leaf sheaths, while relatively lower expression levels are detected in panicles (Figure 4A). Furthermore, it was observed that the expression of *HTS2* was markedly elevated in response to high temperature treatment (45 °C), reaching a peak level of 17.8-fold up-regulation after 3 h (Figure 4B). In addition, the expression profiles of *HTS2* were examined under different plant hormones treatments. The results of qRT-PCR showed that *HTS2* expression was strongly upregulated by salicylic acid (SA) treatment, reaching a 3.4-fold increase at 1 h, whereas no discernible alteration was observed in response to abscisic acid (ABA), auxin (IAA), and jasmonic acid (JA) treatments (Figure 4C).

### 2.4. The Heat-Sensitive Phenotype of hts2 Can Be Restored by Serotonin Complementation

**Figure 5:**
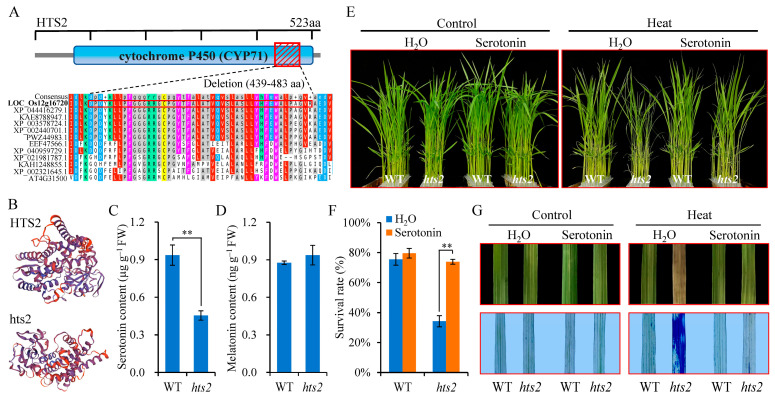
Biochemical complementation of *hts2*. (**A**) Conserved domain architectures of *HTS2* protein derived from the Pfam database (http://pfam.xfam.org/ (accessed on 13 December 2024)). Red box shows the site of the 45 aa (amino acid) deletion from 439 to 483 aa. The amino acid sequence of a conserved region around the mutation site is aligned with similar regions of *HTS2* homologous proteins from different plant species. The alignment was generated with the MAFFT (https://mafft.cbrc.jp/alignment/software/ (accessed on 18 July 2022)). Residues are color coded according to their conservancy. LOC_Os12g16720 (*Oryza sativa*; OsHTS2); XP_044416279.1 (*Triticum aestivum*); KAE8788947.1 (*Hordeum vulgare*); XP_003578724.1 (*Brachypodium distachyon*); XP_002440701.1 (*Sorghum bicolor*); PWZ44983.1 (*Zea mays*); EEF47566.1 (*Ricinus communis*); XP_040959729.1 (*Gossypium hirsutum*); XP_021981787.1 (*Helianthus annuus*); KAH1248855.1 (*Glycine max*); XP_002321645.1 (*Populus trichocarpa*); AT4G31500 (*Arabidopsis thaliana*). (**B**) Three-dimensional models of *HTS2* and mutant protein predicted using SWISS-MODEL. (**C**,**D**) Changes in serotonin (**C**) and melatonin (**D**) contents in leaves of the wild type (WT) and *hts2* mutant plants. Data are means ± SD (n = 5). Asterisks indicate significant difference between the WT and mutant by Student’s *t*-tests (** *p* < 0.01). (**E**) Heat stress phenotypes of 2-week-old WT and *hts2* mutant seedlings treated with or without 100 μM serotonin. (**F**) Survival rates of WT and *hts2* mutant seedlings following heat treatment as described in (**E**). Data are means ± SD (n = 3 replicates, 30–40 individual seedlings per replicate). Asterisks indicate significant difference between treatments by Student’s *t*-tests (** *p* < 0.01). (**G**) Heat stress phenotypes of WT and *hts2* mutant as examined with detached leaves with or without 100 μM serotonin supplementation. The lower panel of (**G**) shows Evans’ blue staining of these heat-challenged detached leaves.

The *HTS2* ORF (1572 bp) codes for a 523 amino acid (aa) protein with a predicted molecular weight of 57.8 kD. *HTS2* contains a conserved domain of cytochrome P450 superfamily (CYP71P1) at 72–503 aa (Figure 5A). This protein was previously identified as an endoplasmic reticulum (ER)-localized sekiguchi lesion (SL) protein that catalyzes the conversion of tryptamine to serotonin [38]. Amino acid alignment analysis revealed conserved sequence similarity for *HTS2s* across representative monocotyledons species, but showed certain differences in the sequence between monocotyledons and dicotyledons species (Figure 5A and Figure S5). Further protein 3D structure prediction suggests that a 45-amino acids deletion (439–483 aa) in the conserved C-terminal domain of cytochrome P450 may alter the normal tertiary structure of the *HTS2* protein (Figure 5B), potentially affecting its enzymatic activity. To test this, we examined whether the serotonin biosynthesis was impaired in the *hts2* mutant. The results of the liquid chromatography-mass spectrometry (LC-MS) analysis indicated a significant reduction in serotonin content in the *hts2* mutant relative to the WT (Figure 5C). Additionally, we measured melatonin levels in WT and *hts2* mutants. The results showed that there was no significant difference in melatonin content between the WT and *hts2* mutant (Figure 5D).

We next investigated whether the heat-sensitive phenotype observed in *hts2* was a consequence of the reduction in serotonin levels. Exogenous serotonin (100 µM) was added to the culture solution to examine whether this could rescue the heat-sensitive phenotype of *hts2* mutant plants. Under normal conditions, no visible phenotypic differences were observed between the *hts2* mutant and WT seedlings following serotonin treatment. When exposed to a temperature of 45 °C, the *hts2* mutant seedlings exhibited heat stress-sensitive phenotypes. However, the addition of 100 µM serotonin to the culture solution completely rescued the heat-sensitive phenotype of the *hts2* mutant compared with WT, as evidenced by both young seedlings and detached leaves (Figure 5E–G). These findings collectively highlight the critical role of *HTS2*-mediated serotonin biosynthesis in regulating rice heat stress responses.

### 2.5. HTS2 Deficiency Accelerates Heat Stress-Induced ROS Accumulation and Cell Death

**Figure 6:**
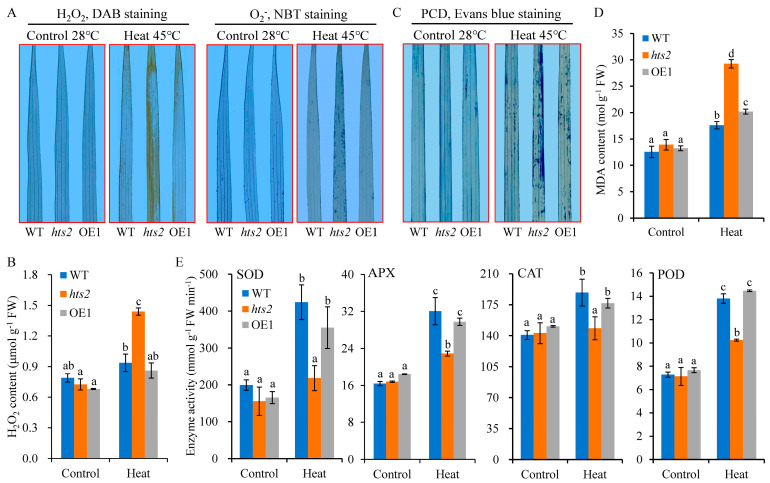
*hts2* mutants accumulated more ROS under heat treatment. (**A**) H_2_O_2_ and O_2_^−^ accumulation revealed by DAB and NBT staining in leaves of 2-week-old wild type (WT), *hts2* and *HTS2* overexpression (OE1) seedlings grown at 28 °C and subjected to heat (45 °C) for 0 and 48 h. (**B**) Determination of H_2_O_2_ contents in leaves of WT, *hts2* and OE plants under normal and heat treatment as described in (**A**). (**C**) Visualization of programmed cell death (PCD) using Evans blue staining in leaves of 2-week-old WT, *hts2* and OE seedlings under normal and heat treatment as described in (**A**). (**D**,**E**) Determination of MDA contents (**D**) and antioxidant enzyme activities (**E**) in leaves of WT, *hts2* and OE seedlings under normal and heat treatment as described in (**A**). Data are means ± SD (n = 3). Different lowercase letters above the error bars indicate a significant difference at *p* < 0.05 by Duncan’s multiple range test.

We next examined whether the *HTS2* mutation influences the ROS accumulation in response to heat stress. Both histochemical staining and quantitative analysis revealed no notable discrepancy of ROS generation between the WT and *hts2* mutant under normal growth conditions. However, the *hts2* leaves exhibited considerably elevated levels of H_2_O_2_ and O_2_^−^ accumulation following exposure to 45 °C heat treatment (Figure 6A,B). Excessive ROS accumulation can induce oxidative stress and results in cellular damage [39]. As expected, staining with Evans blue showed more dead cells in *hts2* leaves compared to the WT after exposure to 45 °C heat treatment, as indicated by the presence of strong dark blue spots in the mutant leaves (Figure 6C). Meanwhile, the malondialdehyde (MDA) levels, a widely used biomarker of lipid oxidation caused by ROS accumulation, were significantly elevated in *hts2* leaves following 45 °C heat treatment (Figure 6D). We also found that heat treatment at 45 °C markedly increased the activity of antioxidant enzymes, including SOD, peroxidase (POD), ascorbate peroxidase (APX), and CAT, in WT leaves, but only slight increase were observed in *hts2* mutant leaves (Figure 6E). Moreover, the levels of both ROS and cell death were fully restored in *HTS2*-overexpression transgenic plants.

**Figure 7:**
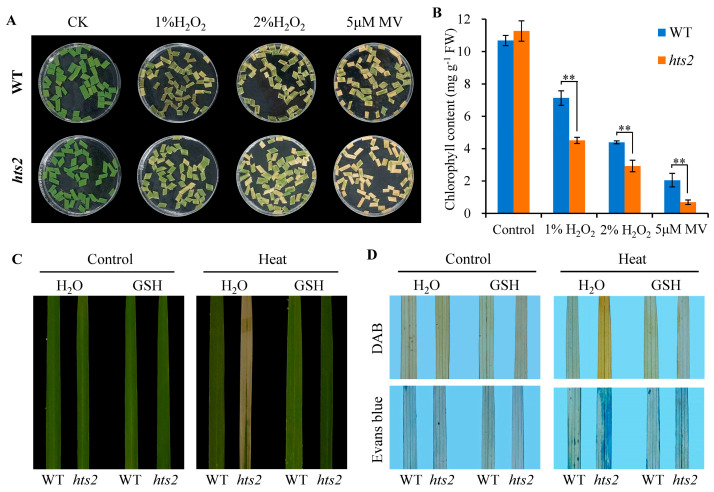
Increased oxidative sensitivity of the *hts2* mutant. (**A**) Effect of different concentrations of H_2_O_2_ and methyl viologen (MV) on detached leaves from the wild type (WT) and *hts2* mutant seedlings. (**B**) Determination of chlorophyll contents in detached leaves of WT and *hts2* seedlings after H_2_O_2_ and MV treatment. Data are means ± SD (n = 3). Asterisks indicate significant difference between the WT and mutant by Student’s *t*-tests (** *p* < 0.01). (**C**) Heat stress phenotypes of WT and *hts2* mutant detached leaves with or without exogenous glutathione supplementation (400 µM). (**D**) DAB and Evans blue staining of the heat-stressed detached leaves of WT and *hts2* described in (**C**).

To further confirm that *HTS2* mutation enhances the sensitivity to oxidative stress, detached leaf segments of WT and *hts2* mutant seedlings were treated with H_2_O_2_ or methylene violet (MV). The results revealed that *hts2* leaves exhibited more sensitivity to H_2_O_2_ or MV treatment. The detached leaves from the *hts2* mutant showed a more rapid rate of bleaching than those from the WT (Figure 7A). After 3 d treatment with 1% H_2_O_2_, 2% H_2_O_2_ or 5 µM MV, the total chlorophyll content was reduced by 60.0%, 74.1%, and 97.4% in *hts2* leaves, but 33.2%, 58.9%, and 80.8% in the WT leaves, respectively, compared to untreated leaves (Figure 7B). Moreover, heat-induced ROS accumulation, cell death, and leaves chlorosis phenotypes in *hts2* detached leaves were substantially eliminated by the addition of 400 µM exogenous reduced glutathione (GSH), a non-enzymatic antioxidant (Figure 7C,D). Taken together, these results clearly demonstrate that *HTS2* regulates heat tolerance by mediating cellular ROS homeostasis.

### 2.6. HTS2 Modulates the Expression of HsfA2 and Its Downstream Heat-Responsive Genes Under High Temperature Condition

**Figure 8:**
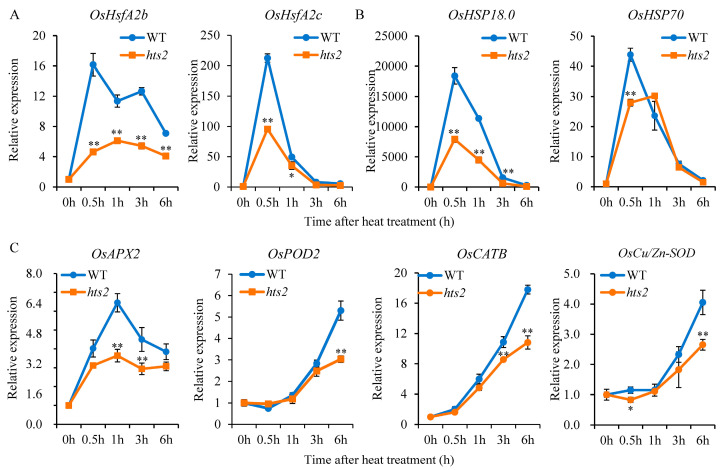
Time course expression pattern of heat stress-responsive genes in the wild type (WT) and *hts2* plants under normal and heat stress condition. qRT-PCR analysis of *HSF* (**A**) and *HSP* (**B**) gene expression, as well as genes involved in subcellular ROS detoxification (**C**), in leaves of 2-week-old WT and *hts2* seedlings exposed to heat treatment (45 °C) for the indicated times. Data are means ± SD (n = 3). Asterisks indicate significant difference between the WT and mutant by Student’s *t*-tests (** *p* < 0.01; * *p* < 0.05).

It has been demonstrated that a heat stress-induced H_2_O_2_ burst plays a critical role in the rapid activation of heat shock genes in plants [3]. We then analyzed the time-dependent expression patterns of previously identified HSFs and downstream heat-responsive genes in the WT and *hts2* mutant seedlings subjected to heat treatment. qRT-PCR analysis showed that 45 °C treatment resulted in a notable elevation in the transcription levels of *HsfA2s* in WT seedlings, reaching a maximum at 0.5 h after heat treatment. However, the heat-activated expression of these *HsfA2s* was markedly diminished in the *hts2* seedlings, particularly the *Hsf2Ab* and *HsfA2c*, which declined to 28.4% and 44.9% of the WT levels, respectively, after 0.5 h heat treatment (Figure 8A). Heat shock proteins (HSPs) and ROS-scavenging enzymes are well-known target genes regulated by HSFs [9]. Our findings revealed a comparable expression pattern of *HSPs* with *HSFs*, whereby the transcription of two detected *HSPs*, *HSP18.0*, and *HSP70*, were significantly suppressed in the *hts2* mutant compared to the WT plants (Figure 8B). Furthermore, we observed that the expression of specific ROS-scavenging genes, such as *OsAPX2*, *OsPOD2*, *OsCATB,* and *OsCu/Zn-SOD*, were significantly up-regulated in both the WT and *hts2* mutant plants under 45 °C treatment. However, the mRNA levels of these genes were notably lower in the *hts2* mutant relative to the WT during the treatment (Figure 8C). These findings indicate that *HTS2* deficiency affects the transcriptional activation of *HSFs* and their downstream targets genes under heat stress, which likely contributes to the vulnerability of the *hts2* mutant to high temperature stress.

## 3. Discussion

Heat stress tolerance in plants is a complex trait governed by a high-complex genetic regulatory network, involving genes responsible for stress sensing, signal transduction, and downstream metabolic responses [1]. In rice, several genes regulating heat tolerance have been identified over the past decade, elucidating a portion of the mechanism underlying the synergistic regulation of heat tolerance [13,40]. We recently showed that *High Temperature Sensitive 1* (*HTS1*), a β-ketoacyl carrier protein reductase functioning in fatty acid biosynthesis, regulates heat tolerance by modulating membrane stability, chloroplast integrity, and stress signaling [41]. However, our understanding of the heat stress response in rice remains largely unknown. In the current study, we demonstrate that *HTS2*, encoding a previously reported cytochrome P450 protein CYP71P1 (designated *SL*; [38]), confers heat stress tolerance in rice by mediating ROS homeostasis and stress-responsive signaling pathways.

Previous studies have demonstrated that *SL*/*HTS2* is a pleiotropic gene that exerts influence over diverse aspects of plant growth and development. For instance, *SL* is involved in the biosynthesis of serotonin, the loss-of-function of *SL* affects plant innate immunity response and causes lesion mimic phenotype [38]. Recent reports showed that allelic mutants of *SL* induce the accumulation of ROS at the lesion sites in leaves, which in turn causes chloroplast degradation, cell death, and premature leaf senescence [42,43]. In this context, we characterized *hts2*, a new allelic mutant of *SL*, for its role in rice heat stress tolerance. In addition to the lesion-mimic and premature senescence phenotypes, *hts2* mutant also exhibits heat-sensitive phenotypes in the seedling stage (Figure 1, Figures S1 and S2). Transcript analysis showed that high temperature treatment significantly upregulated *HTS2* expression (Figure 5). Compared with the normal temperature condition (28 °C), *hts2* mutant seedlings accumulate higher contents of ROS and exhibited more pronounced cell death at elevated temperature (45 °C) than the WT (Figure 6). Additionally, the formation of necrotic spots in *hts2* leaves occurred earlier under high temperature treatment compared to the WT (Figure 2). Further biochemical and physiological analyses suggested that the positive role of *HTS2* in heat stress tolerance is associated with its function in modulating serotonin biosynthesis. The *hts2* mutant exhibits reduced serotonin accumulation compared to the WT, and the heat-sensitive phenotype of the *hts2* mutant can be fully restored by applying exogenous serotonin (Figure 4). These results suggest the critical role of *HTS2* in serotonin biosynthesis and heat stress adaptation in rice.

Oxidative burst is a ubiquitous early event in plants subjected to abiotic stress, leading to oxidative cell damage and diminished heat tolerance [15]. Serotonin is a well-known natural antioxidant in mammals, exhibiting strong in vitro antioxidant activity and playing a critical role in scavenging free radicals in different cell types [44,45]. Recent evidence suggests that serotonin may also function as a potential antioxidant in plants. In rice, serotonin accumulation alleviates biotic stress by acting as a scavenger of oxygen radicals, thereby protecting uninfected tissues from oxidative damage induced by the hypersensitive response [34]. Additionally, elevated endogenous serotonin levels delay leaf senescence by efficiently scavenging ROS in senescent tissues, and vice versa [46]. In this study, we demonstrated that the serotonin-deficient mutant *hts2* exhibits more severe oxidative damage under heat stress, as evidenced by significantly higher ROS accumulation, increased membrane lipid peroxidation, and elevated cell death in comparison to the WT (Figure 6A–D). Moreover, the introduction of exogenous oxidants (H_2_O_2_ or MV) markedly accelerated the levels of oxidative damage in the *hts2* mutant when compared to the WT (Figure 7A,B). The reduced antioxidant capacity of the *hts2* mutant was further evidenced by the application of exogenous antioxidants (e.g., GSH), which effectively reversed the elevated ROS accumulation and heat-sensitive phenotype observed in the *hts2* mutant (Figure 7C,D). These findings indicate that plant resistance to heat stress relies on abundant serotonin synthesis to maintain ROS homeostasis.

Serotonin serves as a precursor for melatonin biosynthesis, and both molecules exhibit functional pleiotropy, playing significant roles in plant growth and development [47]. Studies have demonstrated that serotonin and melatonin are functionally correlated in various plant processes, including stress responses [25]. For example, both compounds help maintain oxidative balance by scavenging stress-induced ROS accumulation, thereby enhancing plant stress tolerance [26]. Thus, the induced synthesis of serotonin and its subsequent conversion to melatonin are critical for mitigating stress-induced oxidative damage in plants [29,47]. However, it has also been shown that serotonin inhibits primary root growth by modulating ROS distribution, likely independently of its conversion into melatonin [48]. In addition, previous studies have indicated that serotonin and melatonin biosynthesis are not synergistically linked. Modulating serotonin biosynthesis by regulating the expression of a key serotonin biosynthesis gene (*T5H*) did not result in a corresponding change in melatonin levels, instead, melatonin accumulation exhibited an opposite trend to serotonin levels [49,50], suggesting that melatonin biosynthesis may not be directly proportional to serotonin levels in plants [27]. Similar to these findings, our data revealed no significant difference in melatonin content between the *hts2* mutant and WT plants (Figure 5D). This may be attributed to the substantially higher abundance of serotonin compared to melatonin (Figure 5C,D) [25]. Therefore, the reduced serotonin content in *hts2* mutants is likely a primary factor contributing to their decreased antioxidant capacity.

**Figure 9:**
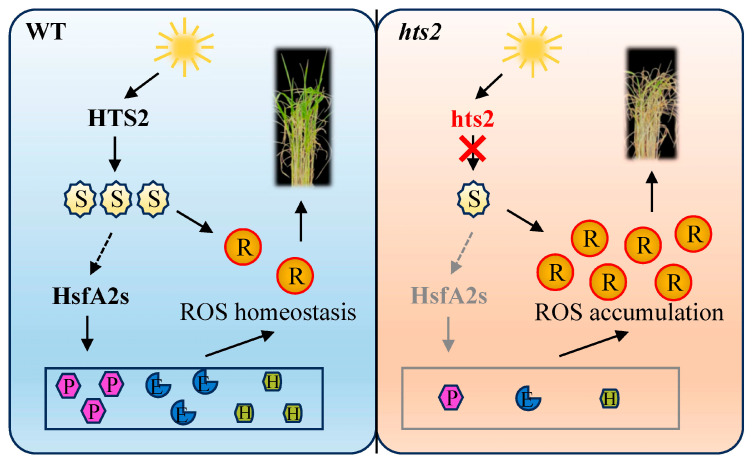
A proposed working model of *HTS2* function in rice heat stress response. The *HTS2* mutation impairs basal serotonin synthesis in rice. The reduced serotonin content in the *hts2* mutant significantly affects heat shock response (HSR), the heat-induced activation of *HsfA2s* and their downstream target genes, such as *HSPs* and antioxidative enzymes, were markedly depressed. This causes low reactive oxygen species (ROS) scavenging capacity, resulting in uncontrolled ROS accumulation and heat damage. Red cross through arrow indicates failure of regulating serotonin synthesis. Dashed arrow indicates indirect regulation of HSFs. S, serotonin; R, ROS; P, heat shock proteins (HSPs); E, ROS scavenging enzymes; H, other HSR genes.

Serotonin is also considered as a signaling molecule that plays important role in the regulation of plant growth as well as stress response [47]. Previous studies suggest that serotonin mediated downstream signaling in *Arabidopsis* root growth primarily operates through ROS generation [48]. Under stress conditions, ROS not only induce oxidative stress but also act as signaling molecules that participating in different signaling cascades, which intersect to regulate plant defense responses [12,39]. It has been established that ROS generation plays a pivotal role in initiating the heat stress signaling cascade that triggers heat stress response [3]. This process is coordinated with the intercellular Ca^2+^ levels and other signaling factors that initiate the activation of *HSFs*-dependent gene networks [9,12]. *HSFA2* is a key regulator within the transcriptional regulatory network governing the heat stress response in plants [51]. It can be activated by *HSFA1*, which in turn induces the expression of a set of downstream genes that are specifically responsive to heat stress [9,41]. Here, we showed a marked reduction in the heat-induced upregulation of *HsfA2* genes in the *hts2* mutant relative to the WT under high temperature conditions (Figure 8A). The repression of heat stress-induced *HSFs* abundance results in a notable reduction in the expression of their downstream targets, including genes encoding HSPs and ROS scavenging enzymes (Figure 8B,C), which are essential for the mitigation of the damage caused by heat stress [7,17]. Consistent with the gene expression results, the activities of these antioxidant enzymes are significantly enhanced in WT plants under high temperature conditions (Figure 6E), aiding in the removal of excess ROS generated during heat stress [52]. However, this heat-induced activity of antioxidant enzymes was largely diminished in the *hts2* mutant (Figure 6E), which may disrupt the balance between ROS generation and scavenging, resulting in excessive ROS accumulation under high temperature conditions. Collectively, we speculated that the reduced serotonin production in the *hts2* mutant might lead to alterations in early ROS burst under heat stress, thereby significantly affecting the heat shock signaling and transcriptional cascades, resulting in excessive ROS accumulation and irreversible heat damage (Figure 9). Further illustration of the crosstalk between serotonin and ROS under heat stress will not only enhance our basic understanding of the mechanism of plant response to heat stress, but also provide strategies to improve rice resilience under high temperature conditions.

## 4. Materials and Methods

### 4.1. Plant Materials and Growth Conditions

The *hts2* mutant was obtained from an ethyl methanesulfonate (EMS)-mutagenized population of the *Indica* rice cultivar Shuhui 527 (wild type; WT). All rice plants were grown in experimental paddy fields with clay loam soil at the China National Rice Research Institute, Hangzhou, China (30.3° N, 120.2° E), following standard agricultural practice during the rice growing season from June to October. For the field experiment, a randomized completely block design with three replications was implemented, with each plot (1.7 m × 2 m) containing 48 rice seedlings. For morphological analyses, rice plants were transferred from paddy fields to pots, and representative photographs were taken at each corresponding stage. After photography, the plants were not returned to the field. For agronomic trait measurements, at least 15 plants from each plot were randomly selected for analysis at maturity. For hydroponic experiments, the plants were grown in a growth chamber under the conditions of a 14 h day (28 °C)/10 h night (24 °C) photoperiod and 65 ± 5% relative humidity. The nutrient solution used in hydroponic experiments containing 1.14 mM NH_4_NO_3_, 0.80 mM CaCl_2_, 1.32 mM MgSO_4_, 0.41 mM K_2_SO_4_, 0.26 mM NaH_2_PO_4_, 0.025 mM Fe-EDTA, 0.007 mM MnCl_2_, 0.015 mM HBO_3_, 0.06 µM (NH_4_)_6_Mo_7_O_24_, 0.12 µM ZnSO_4,_ and 0.12 µM CuSO_4_. The pH of the nutrient solution was adjusted to 5.8. Heat stress conditions and heat tolerance assays were conducted as described previously [41]. For the exogenous serotonin and reduced glutathione (GSH) supplemental experiments, 2-week-old hydroponic WT and *hts2* mutant seedlings were treated with or without 100 µM serotonin or 400 µM GSH under normal and heat conditions. To investigate the response of *HTS2* to different phytohormones, 2-week-old WT seedlings were treated with 10 µM abscisic acid (ABA), 50 µM methyl jasmonate (MeJA), 10 µM auxin (IAA) or 500 µM SA.

### 4.2. Map-Based Cloning of HTS2

To map the *HTS2* locus, the *hts2* mutant was hybridized with the *Japonica* cultivar Nipponbare to generate an F2 populations. Individuals exhibiting the mutant phenotype from F2 population were selected for genetic mapping using 180 simple sequence repeats (SSRs) and sequence tagged site (STS) markers [53]. For fine mapping, additional STS markers were generated around the *hts2* locus based on genomic polymorphisms between 93-11 (an *Indica* variety) and Nipponbare. Candidate genes were predicted using the public rice databases (the Rice Genome Annotation Project; https://rice.uga.edu/ (accessed on 1 November 2016)). These genes were then amplified both from WT and *hts2* mutant plants and sequenced to identify the *hts2* mutation. All the primers used in this study are listed in Table S1.

### 4.3. Plasmid Construction and Plant Transformation

For the construction of *HTS2* overexpression vectors, full-length coding region of *HTS2* was amplified from cDNA of Nipponbare and the PCR product was inserted into ApaI and XbaI sites of the AHLG, a modified pCAMBIA1300 vector (Figure S7A). For RNA interference (RNAi) construct, the antisense fragment (286-bp) of *HTS2* was amplified from cDNA of Nipponbare and sequentially cloned into the pTCK303 vector as described previously [53] (Figure S7B). The binary constructs were transformed into the *hts2* mutant (overexpression construct) or Nipponbare (RNAi construct) via *Agrobacterium*-mediated transformation method [54]. The full protocol is provided in the Supplementary Materials.

### 4.4. RNA Extraction and Quantitative Real-Time PCR

Total RNA was isolated from rice tissues using TRIzol reagent (Invitrogen, Carlsbad, CA, USA). cDNA was synthesized with the PrimeScript™ RT reagent Kit (TaKaRa, Dalian, China) following the manufacturer’s instructions. Quantitative real-time PCR (qRT-PCR) was conducted using a CFX96 Real-Time PCR Detection System (Bio-Rad, Hercules, CA, USA) with SYBR^®^ Premix Ex Taq™ (TaKaRa) under the following conditions: 95 °C for 30 s, 40 cycles of 95 °C for 5 s, and 60 °C for 10 s. The 2^−ΔΔCT^ method was employed to quantify gene expression levels with three technical replicates for each biological sample. The rice Actin gene (*LOC_Os03g50885*) was used as an internal control.

### 4.5. Cell Physiology Analysis

The accumulation of H_2_O_2_ and O_2_^−^ in the leaves were visualized using 3,3′-diaminobenzidine (DAB) and nitro blue tetrazolium (NBT) staining, respectively, as previously described [55]. Cell death was assessed by trypan blue staining according to [41]. H_2_O_2_ quantification was conducted using Amplex™ Red kit (Invitrogen) following the vendor’s instructions. Fresh leaf samples (0.1 g) were homogenized in 1 mL of 50 μM phosphate buffer (pH 7.4) and centrifuged at 12,000× *g* for 10 min at 4 °C. Aliquots (50 μL) of standard curve samples, controls, and crude extracts were added to a microplate, mixed with 50 μL of working solution (containing 100 μM 10-acetyl-3,7-dihydroxyphenoxazine and 0.2 U mL^−1^ horseradish peroxidase), and incubated at room temperature for 30 min in the dark. The absorbance at 560 nm was measured immediately using a microplate reader. To assess antioxidant enzyme activities, 0.1 g rice leaves were homogenized in 4 mL of 50 mM PBS buffer (potassium phosphate; pH 7.8). The homogenate was centrifuged at 12,000× *g* for 10 min at 4 °C and the supernatant was used for enzyme activities measurement, following our previously described method [55]. MDA content was measured using a commercial kit (Solarbio, Beijing, China) according to the manufacturer’s instructions. Briefly, 0.1 g fresh leaf samples were homogenized in 1 mL extraction buffer and centrifuged at 8000× *g* for 10 min at 4 °C. A 200 µL aliquot of the upper layer was mixed with 800 µL of reaction buffer, boiled for 60 min and immediately cooled on ice. Following centrifugation at 10,000× *g* for 10 min at room temperature, the absorbance of the supernatant was measured at 532 and 600 nm using a spectrophotometer. The MDA content was calculated as 32.258 × (A532-A600)/Fresh Weight.

### 4.6. H_2_O_2_ and Methyl Viologen Treatment

The second fully expanded leaves from 4-week-old seedlings of WT and *hts2* mutant were cut into small segments and placed in sterilized glass dishes containing 20 mL of deionized water (floated, with abaxial surface down) in the absence or presence of methyl viologen (MV; 5 μM) or H_2_O_2_ (1% and 2%; *v*/*v*). The dishes were incubated in continuous light at room temperature until identifiable differences in leaf color were observed. The total chlorophyll content was measured after incubation according to the method described previously [53].

### 4.7. Quantification of Serotonin and Melatonin Contents

For serotonin quantification, WT and *hts2* mutant seedlings were grown in nutrient solution for two weeks. Fresh leaf tissues were collected, ground into a fine powder using liquid nitrogen, and weighed (0.2 g). The samples were extracted with 1 mL methanol, vortexed for 20 min, and centrifuged twice at 12,000× *g* for 15 min at 4 °C. The supernatants were lyophilized to dryness, resuspended with 500 µL of methanol/water (1/1, *v*/*v*), and filtered through a 0.22 μm membrane. The serotonin content was analyzed using liquid chromatography-tandem mass spectrometry (LC-MS/MS) according to previous reports [56,57]. For melatonin determination, fresh leaf tissue was homogenized in 50 mM PBS (pH 7.4) at a ratio of 1:9 (*w*/*v*) and centrifuged at 3000× *g* for 20 min at 4 °C. The supernatant was collected, and melatonin levels were analyzed using a commercial ELISA kit (Mlbio, Shanghai, China) following the manufacturer’s instructions.

### 4.8. Statistical Analysis

Statistical analyses were conducted using Microsoft Excel 2019 and the Data Processing System (DPS) v16.05 statistical software package, employing Student’s *t*-test and the Duncan’s multiple range test. All experiments were performed at least three times.

## Data Availability

The data used in this study are available within the article and its accompanying Supplementary Materials.

## Supplementary Materials

The following supporting information can be downloaded at: https://www.mdpi.com/article/10.3390/plants14071072/s1, Figure S1: Phenotypic characterization of the *hts2* mutant. Figure S2: Pre-mature phenotype of the *hts2* mutant. Figure S3: Growth morphology of *HTS2*-OE transgenic lines under field conditions. Figure S4: Phenotypic analysis of *HTS2*-RNAi lines. Figure S5: Alignment of amino acid sequences of HTS2 proteins from different plant species. Figure S6: Representative chromatograms of the LC-MS/MS analysis of the samples and the standard serotonin. Figure S7: The *HTS2* over-expression and RNA interference (RNAi) constructs used in this work. Table S1: A list of primers used in this study.

## References

[1] Zhang J.Y., Li X.M., Lin H.X., Chong K. (2019). Crop improvement through temperature resilience. Annu. Rev. Plant Biol..

[2] Eckardt N.A., Ainsworth E.A., Bahuguna R.N., Broadley M.R., Busch W., Carpita N.C., Castrillo G., Chory J., DeHaan L.R., Duarte C.M. (2023). Climate change challenges, plant science solutions. Plant Cell.

[3] Mittler R., Finka A., Goloubinoff P. (2012). How do plants feel the heat?. Trends Biochem. Sci..

[4] Zhang H., Zhu J., Gong Z., Zhu J.K. (2022). Abiotic stress responses in plants. Nat. Rev. Genet..

[5] VanWallendael A., Soltani A., Emery N.C., Peixoto M.M., Olsen J., Lowry D.B. (2019). A molecular view of plant local adaptation: Incorporating stress-response networks. Annu. Rev. Plant Biol..

[6] Li B.J., Gao K., Ren H.M., Tang W.Q. (2018). Molecular mechanisms governing plant responses to high temperatures. J. Integr. Plant Biol..

[7] Bakery A., Vraggalas S., Shalha B., Chauchan H., Benhamed M., Fragkostefanakis S. (2024). Heat stress transcription factors as the central molecular rheostat to optimize plant survival and recovery from heat stress. New Phytol..

[8] Raturi V., Zinta G. (2024). HSFA1 heat shock factors integrate warm temperature and heat signals in plants. Trends Plant Sci..

[9] Ohama N., Sato H., Shinozaki K., Yamaguchi-Shinozaki K. (2017). Transcriptional regulatory network of plant heat stress response. Trends Plant Sci..

[10] Ding Y., Shi Y., Yang S. (2020). Molecular regulation of plant responses to environmental temperatures. Mol. Plant.

[11] He N.Y., Chen L.S., Sun A.Z., Zhao Y., Yin S.N., Guo F.Q. (2022). A nitric oxide burst at the shoot apex triggers a heat-responsive pathway in Arabidopsis. Nat. Plants.

[12] Mittler R., Zandalinas S.I., Fichman Y., Van Breusegem F. (2022). Reactive oxygen species signalling in plant stress responses. Nat. Rev. Mol. Cell Biol..

[13] Kan Y., Mu X.R., Gao J., Lin H.X., Lin Y. (2023). The molecular basis of heat stress responses in plants. Mol. Plant.

[14] Wahid A., Gelani S., Ashraf M., Foolad M.R. (2007). Heat tolerance in plants: An overview. Environ. Exp. Bot..

[15] Choudhury F.K., Rivero R.M., Blumwald E., Mittler R. (2017). Reactive oxygen species, abiotic stress and stress combination. Plant J..

[16] Xu Y.F., Chu C.C., Yao S.G. (2021). The impact of high-temperature stress on rice: Challenges and solutions. Crop J..

[17] Xia S., Liu H., Cui Y., Yu H., Rao Y., Yan Y., Zeng D., Hu J., Zhang G., Gao Z. (2022). UDP-N-acetylglucosamine pyrophosphorylase enhances rice survival at high temperature. New Phytol..

[18] Dumanovic J., Nepovimova E., Natic M., Kuca K., Jacevic V. (2021). The significance of reactive oxygen species and antioxidant defense system in plants: A concise overview. Front. Plant Sci..

[19] Liao M., Ma Z.M., Kang Y.R., Zhang B.M., Gao X.L., Yu F., Yang P.F., Ke Y.G. (2023). ENHANCED DISEASE SUSCEPTIBILITY 1 promotes hydrogen peroxide scavenging to enhance rice thermotolerance. Plant Physiol..

[20] Wang J.J., Xu J., Wang L., Zhou M.Y., Nian J.Q., Chen M.M., Lu X.L., Liu X., Wang Z., Cen J.S. (2023). SEMI-ROLLED LEAF 10 stabilizes catalase isozyme B to regulate leaf morphology and thermotolerance in rice (*Oryza sativa* L.). Plant Biotechnol. J..

[21] Fang Y., Liao K., Du H., Xu Y., Song H., Li X., Xiong L. (2015). A stress-responsive NAC transcription factor SNAC3 confers heat and drought tolerance through modulation of reactive oxygen species in rice. J. Exp. Bot..

[22] Qiao B., Zhang Q., Liu D.L., Wang H.Q., Yin J.Y., Wang R., He M.L., Cui M., Shang Z.L., Wang D.K. (2015). A calcium-binding protein, rice annexin OsANN1, enhances heat stress tolerance by modulating the production of H_2_O_2_. J. Exp. Bot..

[23] Cui Y., Lu S., Li Z., Cheng J., Hu P., Zhu T., Wang X., Jin M., Wang X., Li L. (2020). CYCLIC NUCLEOTIDE-GATED ION CHANNELs 14 and 16 promote tolerance to heat and chilling in rice. Plant Physiol..

[24] Liu X., Ji P., Liao J., Duan X., Luo Z., Yu X., Jiang C.J., Xu C., Yang H., Peng B. (2025). CRISPR/Cas knockout of the NADPH oxidase gene OsRbohB reduces ROS overaccumulation and enhances heat stress tolerance in rice. Plant Biotechnol. J..

[25] Mishra V., Sarkar A.K. (2023). Serotonin: A frontline player in plant growth and stress responses. Physiol. Plant.

[26] Kaur H., Mukherjee S., Baluska F., Bhatla S.C. (2015). Regulatory roles of serotonin and melatonin in abiotic stress tolerance in plants. Plant Signal Behav..

[27] Sun C., Liu L., Wang L., Li B., Jin C., Lin X. (2021). Melatonin: A master regulator of plant development and stress responses. J. Integr. Plant Biol..

[28] Dharmawardhana P., Ren L., Amarasinghe V., Monaco M., Thomason J., Ravenscroft D., McCouch S., Ware D., Jaiswal P. (2013). A genome scale metabolic network for rice and accompanying analysis of tryptophan, auxin and serotonin biosynthesis regulation under biotic stress. Rice.

[29] Mukherjee S., David A., Yadav S., Baluska F., Bhatla S.C. (2014). Salt stress-induced seedling growth inhibition coincides with differential distribution of serotonin and melatonin in sunflower seedling roots and cotyledons. Physiol. Plant.

[30] Erland L.A., Turi C.E., Saxena P.K. (2016). Serotonin: An ancient molecule and an important regulator of plant processes. Biotechnol. Adv..

[31] Akcay U.C., Okudan N. (2023). Exogenous serotonin improves drought and salt tolerance in tomato seedlings. Plant Growth Regul..

[32] Wan J., Zhang P., Wang R., Sun L., Ju Q., Xu J. (2018). Comparative physiological responses and transcriptome analysis reveal the roles of melatonin and serotonin in regulating growth and metabolism in Arabidopsis. BMC Plant Biol..

[33] Li J.J., Zhang H.Y., Yue D.F., Chen S.Y., Yin Y.X., Zheng C.F., Chen Y. (2024). Endogenous serotonin induced by cold acclimation increases cold tolerance by reshaping the MEL/ROS/RNS redox network in Kandelia obovata. J. For. Res..

[34] Hayashi K., Fujita Y., Ashizawa T., Suzuki F., Nagamura Y., Hayano-Saito Y. (2016). Serotonin attenuates biotic stress and leads to lesion browning caused by a hypersensitive response to *Magnaporthe oryzae* penetration in rice. Plant J..

[35] Kumar G., Saad K.R., Arya M., Puthusseri B., Mahadevappa P., Shetty N.P., Giridhar P. (2021). The synergistic role of serotonin and melatonin during temperature stress in promoting cell division, ethylene and isoflavones biosynthesis in *Glycine max*. Curr. Plant Biol..

[36] Liang C., Wang Y., Zhu Y., Tang J., Hu B., Liu L., Ou S., Wu H., Sun X., Chu J. (2014). OsNAP connects abscisic acid and leaf senescence by fine-tuning abscisic acid biosynthesis and directly targeting senescence-associated genes in rice. Proc. Natl. Acad. Sci. USA.

[37] Qiu T., Zhao X., Feng H., Qi L., Yang J., Peng Y.L., Zhao W. (2021). OsNBL3, a mitochondrion-localized pentatricopeptide repeat protein, is involved in splicing nad5 intron 4 and its disruption causes lesion mimic phenotype with enhanced resistance to biotic and abiotic stresses. Plant Biotechnol. J..

[38] Fujiwara T., Maisonneuve S., Isshiki M., Mizutani M., Chen L., Wong H.L., Kawasaki T., Shimamoto K. (2010). Sekiguchi lesion gene encodes a cytochrome P450 monooxygenase that catalyzes conversion of tryptamine to serotonin in rice. J. Biol. Chem..

[39] Singh V.P., Jaiswal S., Wang Y., Feng S., Tripathi D.K., Singh S., Gupta R., Xue D., Xu S., Chen Z.H. (2024). Evolution of reactive oxygen species cellular targets for plant development. Trends Plant Sci..

[40] Xing Y.H., Lu H.Y., Zhu X.F., Deng Y.F., Xie Y.J., Luo Q.H., Yu J.S. (2024). How rice responds to temperature changes and defeats heat stress. Rice.

[41] Chen F., Dong G., Wang F., Shi Y., Zhu J., Zhang Y., Ruan B., Wu Y., Feng X., Zhao C. (2021). A beta-ketoacyl carrier protein reductase confers heat tolerance via the regulation of fatty acid biosynthesis and stress signaling in rice. New Phytol..

[42] Cui Y., Peng Y., Zhang Q., Xia S., Ruan B., Xu Q., Yu X., Zhou T., Liu H., Zeng D. (2021). Disruption of EARLY LESION LEAF 1, encoding a cytochrome P450 monooxygenase, induces ROS accumulation and cell death in rice. Plant J..

[43] Zheng Y., Xu J., Wang F., Tang Y., Wei Z., Ji Z., Wang C., Zhao K. (2021). Mutation types of CYP71P1 cause different phenotypes of mosaic spot lesion and premature leaf senescence in rice. Front. Plant Sci..

[44] Azouzi S., Santuz H., Morandat S., Pereira C., Cote F., Hermine O., El Kirat K., Colin Y., Le Van Kim C., Etchebest C. (2017). Antioxidant and membrane binding properties of serotonin protect lipids from oxidation. Biophys. J..

[45] Azmitia E.C. (2007). Serotonin and brain: Evolution, neuroplasticity, and homeostasis. Int. Rev. Neurobiol..

[46] Kang K., Kim Y.S., Park S., Back K. (2009). Senescence-induced serotonin biosynthesis and its role in delaying senescence in rice leaves. Plant Physiol..

[47] Mukherjee S. (2018). Novel perspectives on the molecular crosstalk mechanisms of serotonin and melatonin in plants. Plant Physiol. Biochem..

[48] Pelagio-Flores R., Ruiz-Herrera L.F., Lopez-Bucio J. (2016). Serotonin modulates Arabidopsis root growth via changes in reactive oxygen species and jasmonic acid-ethylene signaling. Physiol. Plant.

[49] Park S., Lee K., Kim Y.S., Back K. (2012). Tryptamine 5-hydroxylase-deficient Sekiguchi rice induces synthesis of 5-hydroxytryptophan and N-acetyltryptamine but decreases melatonin biosynthesis during senescence process of detached leaves. J. Pineal Res..

[50] Park S., Byeon Y., Back K. (2013). Transcriptional suppression of tryptamine 5-hydroxylase, a terminal serotonin biosynthetic gene, induces melatonin biosynthesis in rice (*Oryza sativa* L.). J. Pineal Res..

[51] Charng Y.Y., Liu H.C., Liu N.Y., Chi W.T., Wang C.N., Chang S.H., Wang T.T. (2007). A heat-inducible transcription factor, HsfA2, is required for extension of acquired thermotolerance in Arabidopsis. Plant Physiol..

[52] Wang P., Liu W.C., Han C., Wang S., Bai M.Y., Song C.P. (2024). Reactive oxygen species: Multidimensional regulators of plant adaptation to abiotic stress and development. J. Integr. Plant Biol..

[53] Chen F., Dong G.J., Ma X.H., Wang F., Zhang Y.L., Xiong E.H., Wu J.H., Wang H.Z., Qian Q., Wu L.M. (2018). UMP kinase activity is involved in proper chloroplast development in rice. Photosynth. Res..

[54] Toki S., Hara N., Ono K., Onodera H., Tagiri A., Oka S., Tanaka H. (2006). Early infection of scutellum tissue with Agrobacterium allows high-speed transformation of rice. Plant J..

[55] Chen F., Wang F., Wu F., Mao W., Zhang G., Zhou M. (2010). Modulation of exogenous glutathione in antioxidant defense system against Cd stress in the two barley genotypes differing in Cd tolerance. Plant Physiol. Biochem..

[56] Lu H.P., Luo T., Fu H.W., Wang L., Tan Y.Y., Huang J.Z., Wang Q., Ye G.Y., Gatehouse A.M.R., Lou Y.G. (2018). Resistance of rice to insect pests mediated by suppression of serotonin biosynthesis. Nat. Plants.

[57] Wang L., Lu H., Zhang X., He Y., Zhang J., Guo X., Fu H., Ye G., Shu Q. (2023). Disruption of serotonin biosynthesis increases resistance to striped stem borer without changing innate defense response in rice. J. Pineal Res..

