# Supplementary material for: Rice Cytochrome P450 Protein CYP71P1 Is Required for Heat Stress Tolerance by Regulating Serotonin Biosynthesis and ROS Homeostasis
Source: Plants (Basel). 2025 Apr 1;14(7):1072. doi: 10.3390/plants14071072 (PMC11990548; doi:10.3390/plants14071072)
Supplement: Supplementary file 1 [file plants-14-01072-s001.zip › plants-3491118-supplementary.pdf]

Rice cytochrome P450 protein CYP71P1 is required for heat stress tolerance by regulating serotonin biosynthesis and ROS homeostasis

Xuantong Lv[1†], Xunan Zhao[1†], Fang Wang[2], Haili Wang[1], Yanli Zhang[1], Banpu Ruan[1], Guojun Dong[3], Yanchun Yu[1], Limin Wu[1*], Fei Chen[1*]

[1]College of Life and Environmental Sciences, Hangzhou Normal University, Hangzhou 311121, China

[2]Institute of Insect Sciences, Zhejiang University, Hangzhou 310058, China

[3]State Key Laboratory for Rice Biology, China National Rice Research Institute, Hangzhou 310006, China

[†]These authors contributed equally to this paper.

[*]Author for correspondence:

Fei Chen (chenfei@hznu.edu.cn); Limin Wu (lmwu2006@aliyun.com)

Contents:

Supplementary Materials and Methods.

Figure S1. Phenotypic characterization of the *hts2* mutant.

Figure S2. Pre-mature phenotype of the *hts2* mutant.

Figure S3. Growth morphology of *HTS2*-OE transgenic lines under field conditions.

Figure S4. Phenotypic analysis of *HTS2*-RNAi lines.

Figure S5. Alignment of amino acid sequences of HTS2 proteins from different plant species.

Figure S6. Representative chromatograms of the LC-MS/MS analysis of the samples and the standard serotonin.

Figure S7. The *HTS2* over-expression and RNA interference (RNAi) constructs used in this work.

Table S1. A list of primers used in this study.

Supplementary Materials and Methods

- **Rice transformation via *Agrobacterium*-mediated method**

Rice transgenic lines were generated using an *Agrobacterium*-mediated transformation method following a previously published protocol [1] with minor modifications. Healthy mature seeds were dehulled and surface-sterilized by soaking in 70% ethanol for 1 minute (min), followed by three rinses with sterile water. The seeds were then sterilized in 50 ml of 2% sodium hypochlorite solution containing one drop of Tween 20, with shaking at 100 rpm for 15 min, and rinsed five times with sterile water. This sterilization step was repeated using 2% sodium hypochlorite solution without Tween 20. After sterilization, the seeds were air-dried and placed on N6D solid medium (0.4% phytagel; the detailed compositions of all mediums used in this protocol are provided in Reference #1), and cultured at 32°C under continuous light for two weeks to induce callus formation.

The *Agrobacterium* strain GV3101, harboring the binary vector, was cultured on LB (Luria-Bertani) solid medium containing 50 μg/mL kanamycin and incubated in the dark at 28°C for 2-3 days. A single monoclonal colony was inoculated into 5 mL of LB liquid medium supplemented with 50 μg/mL kanamycin and incubated at 28°C on a shaker (250 rpm) for 24 hours. Subsequently, 500 μL of the bacterial culture was transferred into 100 mL of AAM medium containing 50 μg/ml acetosyringone and cultured at 28°C on a shaker (250 rpm) for approximately 10 hours until the OD600 reached 0.1 - 0.3.

Pre-cultured calli were immersed in *Agrobacterium* suspension for 1 min, then placed on a Petri dish lined with sterilized filter paper. After air-drying the surface, the plate was sealed with Parafilm and incubated in the dark at 25°C for 3 days. Following co-cultivation, the calli were rinsed five times with sterile water containing 400 μg/mL carbenicillin. After removing excess water, the calli were transferred to N6D medium supplemented with 400 μg/mL carbenicillin and 50 μg/mL hygromycin B, and cultured under continuous light at 32°C for approximately 15 days.

The proliferated calli were transferred to RE-III medium (differentiation medium) supplemented with 350 μg/mL carbenicillin and 500 μg/mL hygromycin B, and

cultured under continuous light at 32°C for approximately 20 days. The differentiated shoots were then transferred to HF medium containing 200 μg/mL carbenicillin and 50 μg/mL hygromycin B and cultured under the same conditions to induce root formation. Finally, rooted plantlets were analyzed by PCR using specific primers to confirm the presence of transgenes. Positive transformants were transplanted to the greenhouse and grown until harvest.

References

1. Toki, S.; Hara, N.; Ono, K.; Onodera, H.; Tagiri, A.; Oka, S.; Tanaka, H. Early infection of scutellum tissue with *Agrobacterium* allows high-speed transformation of rice. Plant J. 2006, 47, 969-976, doi:10.1111/j.1365-313X.2006.02836.x.

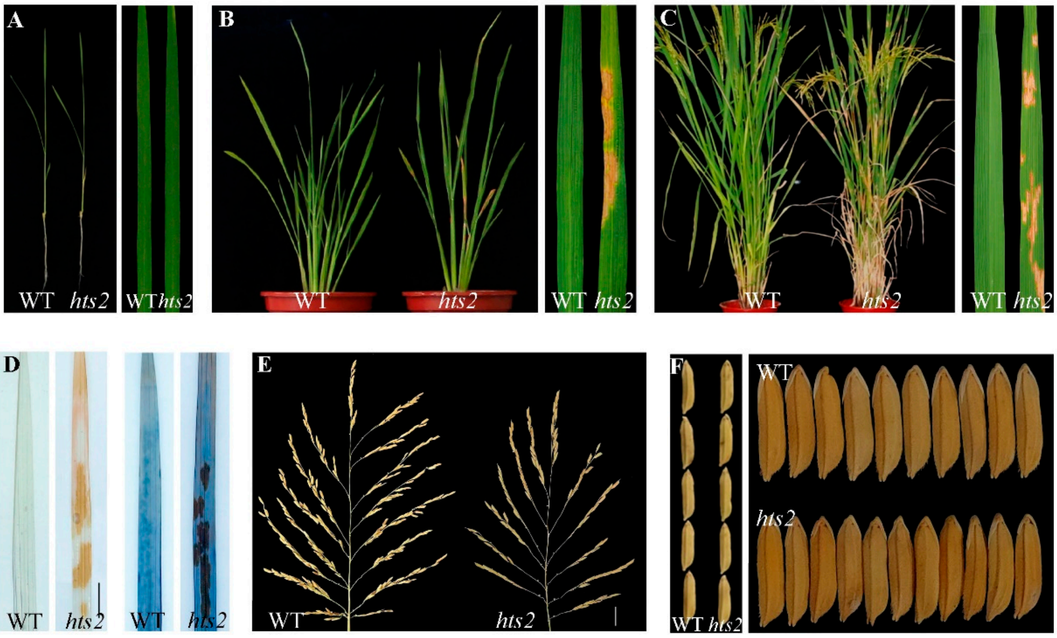

G

Material	Plant height (cm)	Tiller number	Panicle length (cm)	No. PB/panicle	No. SB/panicle	1000-grain weight
WT	129.25±4.3	12.15±4.49	27.77±1.49	13.13±1.06	41.47±11.10	31.74±0.44
hts2	114.94±3.78**	11.83±3.93	23.9±1.8**	11.34±1.2**	27.06±10.32**	32.74±0.8

Figure S1. Phenotypic characterization of the *hts2* mutant. (A-C) Growth phenotype of the wild-type (WT) and *hts2* mutant at seedling stage (A), tillering stage (B) and heading stage (C). Enlarged views of leaves in WT and *hts2* mutant at the corresponding stages are shown (right). Plants were transferred from paddy fields to pots, and representative photographs were taken at each corresponding stage. After photography, the plants were not returned to the field. (D) DAB and NBT staining in leaves of WT and *hts2* mutant. (E) The panicle morphology of WT and *hts2* mutant. (F) Seed size phenotypes of WT and *hts2* mutant. (G) Quantitation of agronomic traits in WT and *hts2* mutant. At maturity, 15 plants from each plot were randomly selected for analysis of agronomic traits. Values are means ± SD. Asterisks indicate significant difference between the WT and mutant by Student's t-tests (**$P < 0.01$).

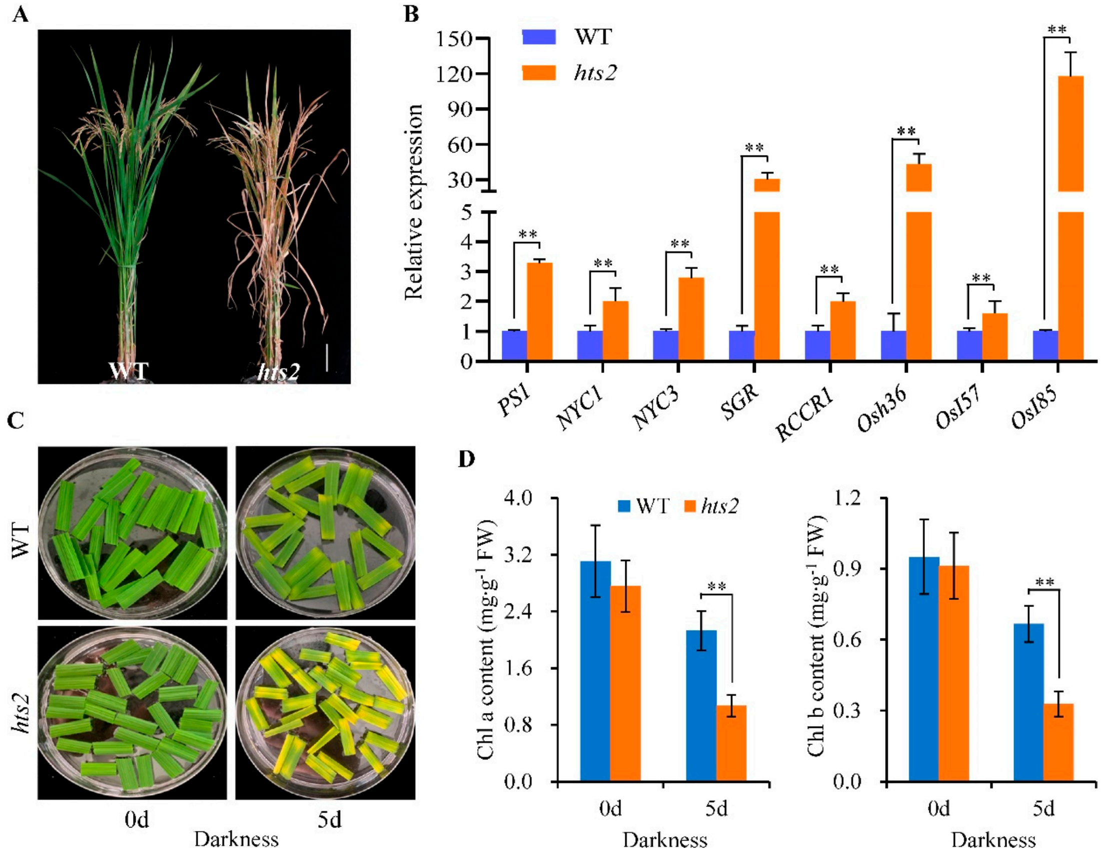

Figure S2. Pre-mature phenotype of the *hts2* mutant. (A) Phenotypes of the wild-type (WT) and *hts2* mutant plants at mature stage (40 day after flowering; Scale bar = 20 cm). (B) Relative expression of genes associated with leaf senescence in WT and *hts2* mutant. (C) Dark-induced leaf senescence in WT and *hts2* mutant. Detached leaf segments from 4-week-old WT and *hts2* mutant seedlings were incubated with water for 5 d in darkness. (D) Chlorophyll content of leaf segments from WT and *hts2* mutant as described in (C). Data are means ± SD (n = 3). Asterisks indicate significant difference between the WT and mutant by Student's t-tests (**P < 0.01). FW, fresh weight.

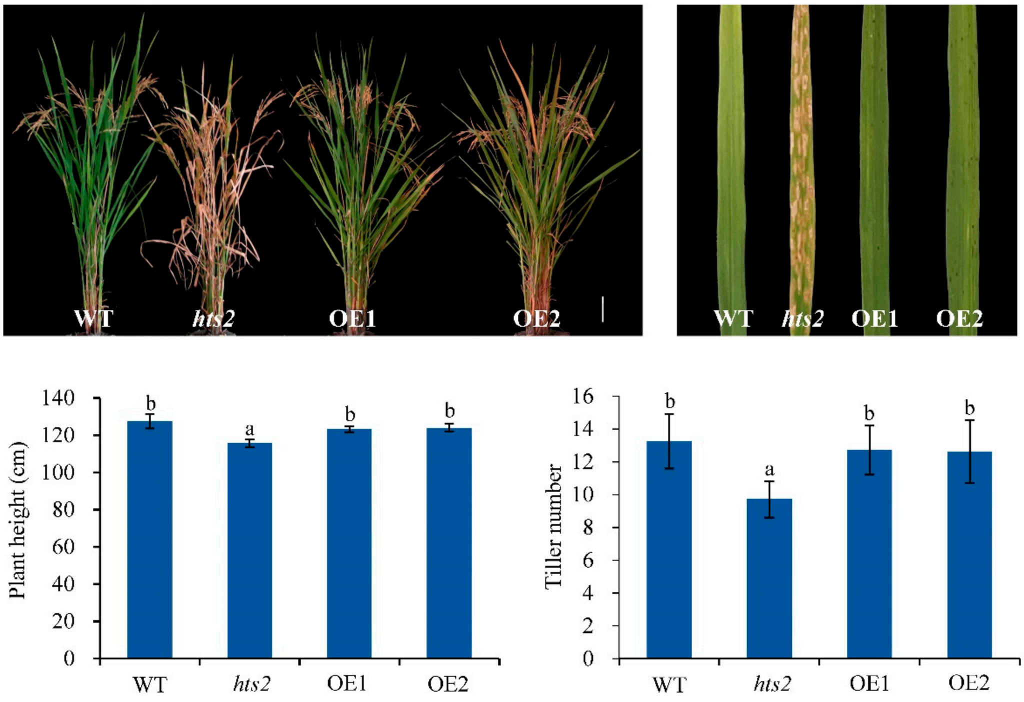

Figure S3. Growth morphology of *HTS2*-OE transgenic lines under field conditions. (A) Morphology of the wild-type (WT), *hts2* and *HTS2*-OE (OE1 and OE2) transgenic lines at the mature stage grown in paddy fields. Bar = 10cm. (B) Leaf phenotypes of WT, *hts2* and *HTS2*-OE lines described in (A). (C, D) Statistics for plant height and tillering number of WT, *hts2* and *HTS2*-OE lines. Data are means ± SD (n = 15). Different lowercase letters above the error bars indicate a significant difference at P < 0.05 by Duncan's multiple range test.

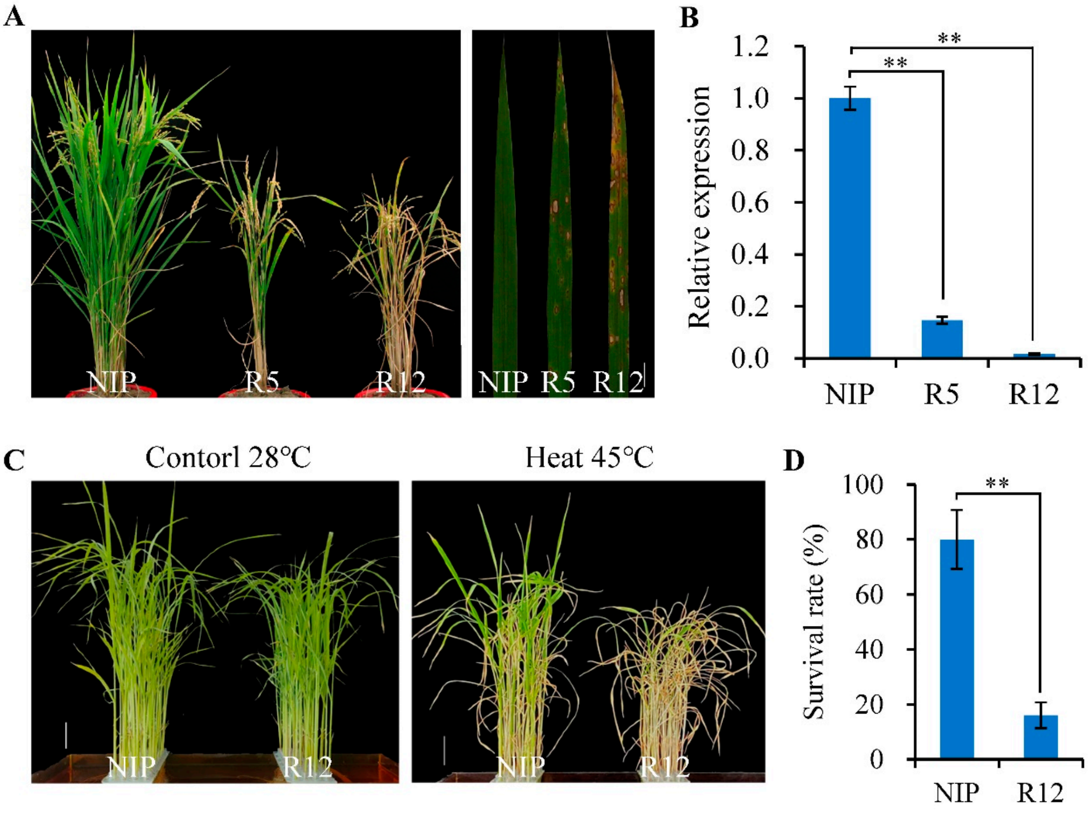

Figure S4. Phenotypic analysis of *HTS2*-RNAi lines. (A) Phenotype comparison of the Nipponbare (NIP, wild-type) plants and two RNAi lines (R5 and R12) at mature stage grown in paddy fields. (B) qRT-PCR analysis of *HTS2* expression in leaves of NIP and RNAi lines. (C) Phenotypes of 2-week-old plants of NIP and RNAi lines grown at 28 °C and after 72h treatment at 45 °C and 2d recovered at 28 °C. (D) Survival rates of NIP and RNAi lines after exposure to heat treatment. Data are means ± SD (n = 3). Asterisks indicate significant difference between the WT and mutant by Student's t-tests (**P < 0.01).

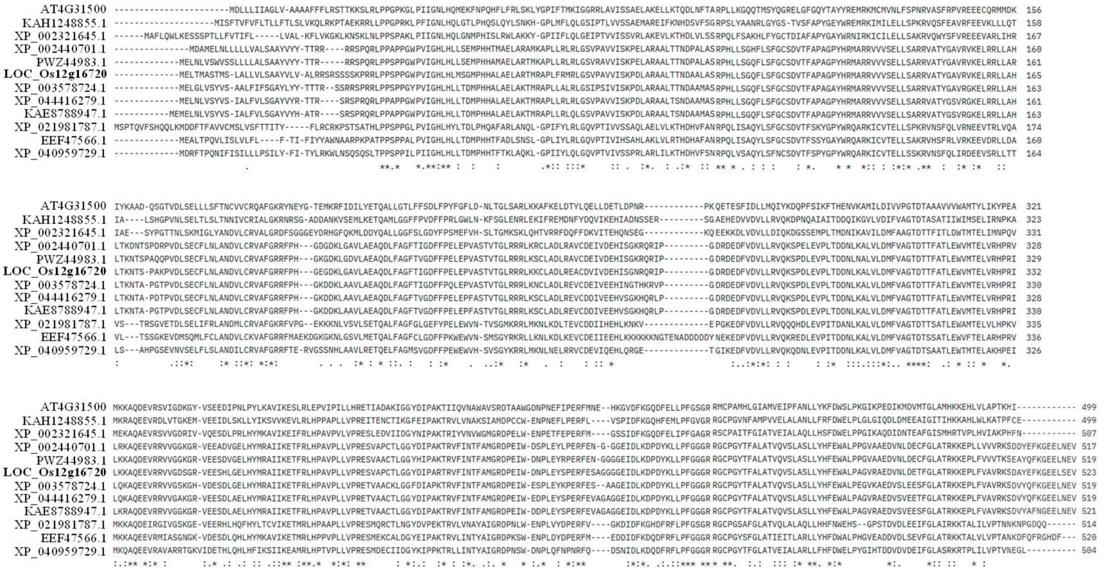

Figure S5. Alignment of amino acid sequences of HTS2 proteins from different plant species. Alignment was created with Clustalo (https://www.ebi.ac.uk/jdispatcher/msa/clustalo/ (accessed on 18 July 2022)). The symbols below each position in the sequence indicate the amount of conservation ('*' exact; ':' conserved substitution; '.' semi-conserved substitution). AT4G31500 (*Arabidopsis thaliana*); KAH1248855.1 (*Glycine max*); XP_002321645.1 (*Populus trichocarpa*); XP_002440701.1 (*Sorghum bicolor*); PWZ44983.1 (*Zea mays*); LOC_Os12g16720 (*Oryza sativa*; OsHTS2); XP_003578724.1 (*Brachypodium distachyon*); XP_044416279.1 (*Triticum aestivum*); KAE8788947.1 (*Hordeum vulgare*); XP_021981787.1 (*Helianthus annuus*); EEF47566.1 (*Ricinus communis*); XP_040959729.1 (*Gossypium hirsutum*).

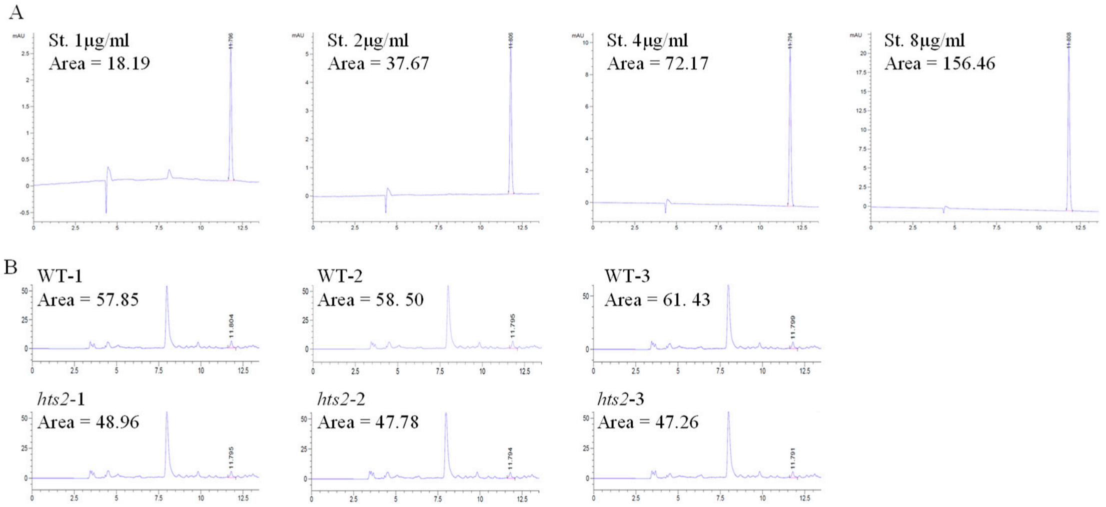

Figure S6. Representative chromatograms of the LC-MS/MS analysis of the samples and the standard serotonin. A. Standard solution with different serotonin concentrations. B. Rice samples with serotonin detected. St. refers to standard. Peak at 11.8 min represents serotonin.

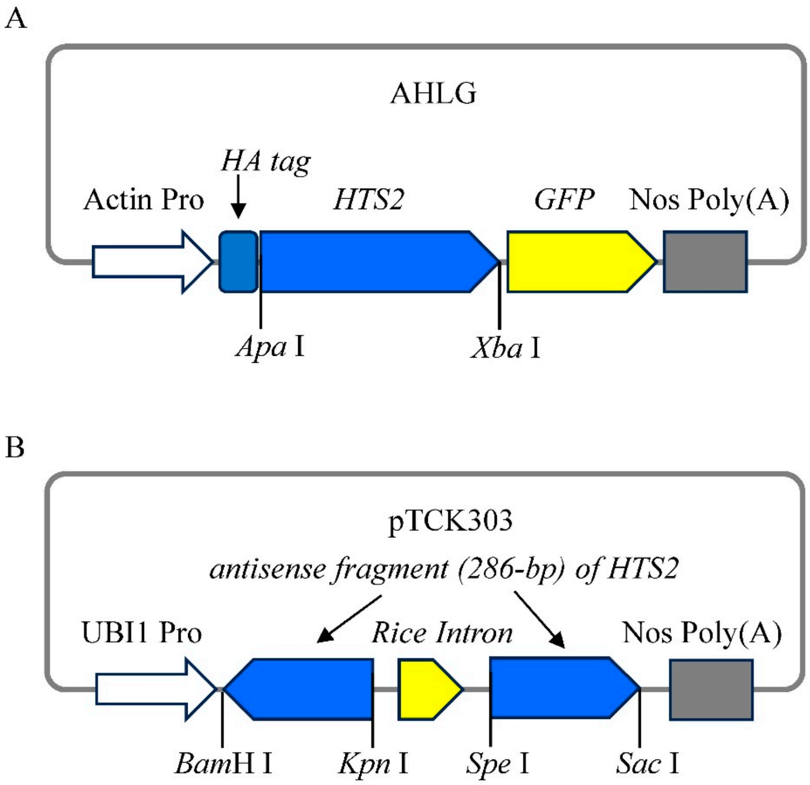

Figure S7. The *HTS2* over-expression and RNA interference (RNAi) constructs used in this work. A. Schematic diagram of the AHLG construct containing the full-length coding region of *HTS2*. B. Construction of RNAi vectors. A 286 bp *HTS2* fragment was ligated into the pTCK303 vector as inverted repeats.

Table S1. A list of primers used in this study.

Primer name	Forword primer sequence (5' → 3')	Reverse primer sequence (5' → 3')
**Map-based cloning**		
YP12-3	ATCAGCAGCAGATTGGTGC	TACCCTTAGTCTCCTATGTGTCC
YP638	CTCGATCCCCTAGCTCTC	TCACCTCGTTCTCGATCC
YP1328	CAGCTTCGGCAAAGCAAAGC	CTCGCTACGTCGACTGATTTGG
YP1437	CATGCTCATGCTTGATGGAG	AGGACAAGAAAGTACATGA
YP1455	AGGTTCAAATGAGACCCAAT	GGTGTTATGTATACGTGGGT
YP1457	CCTTCTCAACCACATCTCAC	GTGGAAGCTCCGAGAAAGTG
YP1459	TAGAAACTATGGTGGCGAG	TGCTAGGTCGATAATCAGG
YP1465	ATTCAGCGCGAGGAGGTGA	CTGTTGGTTTCCTCCATCAG
YP1671	CTGGCTTTACTGTTTGCGAT	TGCACATGCACCTAGGTGCA
**Genotyping**		
YP6087	TGTTCATCAACACGTTCGCCAT	TCGACCAGGATGGGCACCA
**Quantitive real time PCR**		
HTS2	AAGGGAGAGGAGCTTAGTGAGG	AGCTCCAGCCTCCAGGTTACTTAG
OsHsfA2b	TGTTGGTGAGGAGAACGACAAC	CCAGCCGTTGGGCCATTTGAGT
OsHsfA2c	GAGCAGTCAGAGTTGGATGGCA	AATCAACTCTATTTTGGACTAG
OsHsp70	CACAAGAAGAAGGTGGACGC	CGATGATGGGGTTGCAGATG
OsHsp18.0	GAGGAGAAGGAGGAGTCGTG	TCGATGGTCTTGGGCTTCTT
OsAPX2	CCTTCTTCAGCTGCCAAGTG	ATCGGCATTAATCTTTTGCGG
OsPOD2	AGGCTCAACTGCTCCAGGGTCA	TGGCAATAAACCGGACAAGCCCT
OsCATB	GCTTGCTTTCTGCCCAGCGATAAT	AAATAGTTTGGGCCAAGACGGTGC
OsCu/Zn-SOD	CAGATTTCACTAAGCGGGCC	CTTCCTAGGTCATCAGAATCAGCA
PR4	AGCGCATATTGTGCCACATG	GGATACACTTGCCACACGAGTCT
PR5	CAACAGCAACTACCAAGTCGTC	CAAGGTGTCGTTTTATTCATCAAC
PR10	CCCTGCCGAATACGCCTAA	CTCAAACGCCACGAGAATTTG
PS1	CAAGAAGCCGAACGGTTC	GTTAGAGTGGAGCAGCAT
NYC1	CATGCAACACCAACAAAAGG	GACCATTCCAGGAGAAGCAG
NYC3	TGTCGTTGCCATGTGAAGAT	TTGGTCACGCCACAAATCTA
SGR	AGGGGTGGTACAACAAGCTG	GCTCCTTGCGGAAGATGTAG
RCCR1	CGCATTTCCTCATGGAATTT	CTTCTCACGCTGTTTGTCCA
Osh36	GCACGGAGGCGAACGA	TTGAGCGGTAGCACCCATT
OsI57	ACCCTAAAGTAAATGAAGTC	CCTGCTCTTGTCTTGTTA
OsI58	GAGCAACGGCGTGGAGA	GCGGCGGTAGAGGAGATG
**Over expression**		
YP5609	gggcccATGGAGCTCACCATGGC	tctagaAACCTCACTAAGCTCCTCTCCC
**RNAi**		
YP5093	gagctcCCAAGAACACCTCGCCGGCG	actagtTGTTCGTCCACGATCACGTC
YP5094	ggatccCCAAGAACACCTCGCCGGCG	ggtaccTGTTCGTCCACGATCACGTC
